# Reimagining the relationship between economics and health– WHO ‘Health for all’ provisions

**DOI:** 10.1186/s12962-024-00512-9

**Published:** 2024-01-23

**Authors:** Stavros Petrou, Mihajlo Jakovljevic

**Affiliations:** 1 Nuffield Department of Primary Care Health Sciences, University of Oxford, Oxford, UK; 2 UNESCO-TWAS, Trieste, Italy; 3 Shaanxi University of Technology, Hanzhong, China; 4 Department of Global Health Economics and Policy, University of Kragujevac, Kragujevac, Serbia

The recent World Health Organization (WHO) Council on the ‘Economics of Health for All’ calls for a radical shift in economic thinking– in each country, region and globally– to prioritize health for all [1]. Underpinning the WHO Council’s report is an increasing recognition by the WHO and other specialized agencies of the United Nations that the structures embedded within most national economies are yielding poor outcomes and inequalities that are difficult to justify.

The WHO Council’s report calls for nothing less than a complete reimagining of traditionally held views about the relationship between economics and health. It makes 13 policy recommendations across four interrelated pillars, namely valuing health for all, financing health for all, innovating for health for all, and strengthening public capacity for health for all. Notably, the report emphasises the interdependency of health and the economy, positioning health as a cross-cutting lens through which all sectors of the economy should be viewed. This perception aligns with a mainstream health economics viewpoint established many decades ago, when it was first understood that improved population health has a strong positive feedback effect on overall societal productivity [2]. However, rather than solely viewing health care and other sectors as stepping stones for pursuing the ultimate goals of economic growth and maximization of gross domestic product (GDP) [3], regardless of the consequences, the report argues that public sector goals should be framed around maximizing human and planetary well-being, and account for the diverse social foundations that promote equity. This process will inevitably require new metrics. It repositions ‘Health for All’ not only as a fundamental human right, but as the overall goal of public policy that requires a repurposing of the economy. Monetary and fiscal policies at national and supranational levels are routes through which ‘Health for All’ can be achieved, but these will need to be complemented with a meaningful engagement with the private sector.

The urgent tone that permeates the WHO Council’s report is underpinned by a recognition of the opportunity costs of inaction. For example, by avoiding healthcare investments at scale, national health systems end up spending more on the deleterious consequences that result from an unhealthy population. The report cites the work of the non-communicable diseases (NCD) Countdown 2030 Collaborators, which suggested that the US$ 140 billion investment required to reduce global deaths from NCDs by one third would generate a 20-fold economic benefit [4]. Moreover, the report emphasises the need for prevention and intervention across multiple sectors of the economy to achieve health and planetary goals. Reductions in subsidies or other forms of incentives to the oil and gas industries, for example, could reduce the incidence of respiratory conditions that result from air pollution and their adverse clinical and economic sequelae [5].

The reorientation towards ‘Health for All’ proposed by the WHO Council has potential radical implications for researchers undertaking conceptual, methodological or applied work in the economic evaluation and policy-related space. First, and perhaps foremost, it challenges us to rethink the goals and preferred approaches of economic evaluation held by health technology assessment (HTA), pricing and reimbursement authorities, which have tended to provide the frameworks for research undertaken in this space. The goal of maximising health outcomes under conditions of finite resources, where health outcomes have commonly been expressed in terms of quality-adjusted life years (QALYs), is implicitly challenged to encompass a broader set of consequences for all those that are impacted. To some extent, HTA authorities are increasingly recognising the importance of capturing all health effects of interventions and policies, whether these are felt by patients, family members of informal carers [6]. Preference-based quality of life measures that capture impacts on family members and informal carers have also been developed [7]. Less is known, however, about how to aggregate effects across patients, family members and informal carers and the relative social values we should place on patient, family member and informal carer quality of life effects. The WHO Council’s report also challenges us to value *‘human health and well-being, with every person able to prosper physically, mentally and emotionally, and endowed with the capabilities and freedom needed to lead lives of dignity, opportunity and community’* [8]. Arguably, this challenges us to move beyond QALY maximization as a policy goal and encompass broader concepts of value [9]. Capability-based instruments that measure broader outcomes such as attachment, security, enjoyment, role and control may play an important role here [10]. However, if capability-based instruments are to be used to inform health care decision making, then a number of operational concerns remain to be addressed. These include selection of preferred capability-based instruments for application, selection of preference-based value sets that underpin them and derivation of evidence around the cost-effectiveness threshold that should be applied in the decision-making process. As an interim step, multi-stakeholder working groups may be needed to consider how HTA processes could and should reflect broader concepts of value.

The call by the WHO Council to place planetary health as a centerpiece within a new system of measurement and value raises the question of how the environmental effects of the interventions and policies we evaluate should be accounted for. Economists have been noticeably slow in developing new frameworks that capture environmental effects within their evaluations. The WHO’s conceptual framework for the quantification and economic valuation of health outcomes originating from health and non-health climate change mitigation and adaptation action provides a useful starting point [11]. It can potentially be developed to capture the disparate impacts of interventions and policies on metrics of planetary health, which in turn directly or indirectly impact human health and wellbeing. We are not aware of any attempts by health care decision makers to develop their guidance to incorporate environmental impacts into HTA processes. A paucity of expertise, data and resources around environmental considerations, and how they should be accounted for, are contributing factors, but are not the whole story. Some academic researchers are bravely rising to the challenge and highlighting the practical mechanisms through which we might integrate carbon emissions, calculated by environmental life cycle assessment, into HTA [12]. This might be done in a number of different ways depending on the preferred evaluative approaches held by different jurisdictions, although it should be recognised that unlike in most of the Global North and East Asian countries, HTA legislation remains widely underdeveloped across the low and middle income countries (LMICs) of the Global South [13]. Proposed strategies include using carbon emissions as a decision modifier within a multi-criteria decision analysis framework [14] or as a monetized cost within a cost-effectiveness analysis [15] or cost-benefit analysis framework [12]. It is incumbent on decision makers and other stakeholders to clarify the way forward in their respective jurisdictions.

The WHO Council’s report is conspicuous in its call for inputs, such as domestic unpaid labour, voluntary work and informal care, which have traditionally been overlooked in national accounting systems and the calculus of health economic evaluations, to be adequately valued in the new ‘Health for All’ ecosystem [1]. This would inevitably have implications for what we should consider as ‘costs’ within health economic evaluations, and how they should be measured and valued. A particularly radical appeal is made to move away from applying narrow monetary-based measures to valuing unpaid labour.

How should we respond to this call? Shifts in thinking will be required around what inputs we include within our data collection instruments and the descriptors we apply to those inputs. Regardless of the methodological requirements of local HTA authorities, researchers should consider applying approaches that capture the effects of the interventions and policies they are evaluating on unpaid labour. Arguably, this should be extended to other forms of time inputs, such as lost leisure time and education losses, that carry opportunity costs. In many research contexts, there will be a challenge to convince research collaborators of the necessity and value of additional data collection. This is likely to remain the case until the paradigm shift implied by the WHO Council’s report becomes embedded. Moreover, a shift away from application of monetary-based measures to unpaid labour and other forms of time inputs is likely to require a shift away from a ‘one size fits all’ evaluative framework.

A strong theme running through the WHO Council’s report is its emphasis on promoting equity and reducing disparities between individuals, whether by socioeconomic status or other characteristics that embody inequities. As with its other recommendations, little detail is provided on how this could or should be achieved. Attempts to deal with such inequities will remain particularly challenging across the huge emerging market economies driving most of the global GDP growth [16]. This is the case because the BRICS nations (Brazil, Russia, India, China and South Africa) and associated rapidly developing countries (e.g. Indonesia, Pakistan, Nigeria, Mexico) count for the vast majority of middle-class growth worldwide, driving global demand for medical goods and services [17]. Yet this is largely limited to their wealthy industrial and coastal megacities with heavily neglected rural and suburban development and poor networks of healthcare facilities [18]. These legacies of the colonial and cold war eras limit affordability of even essential pharmaceuticals and medical devices amongst hundreds of millions of people across Asia, Africa and Latin America [19]. Such a landscape is illustrated by metrics such as high Gini coefficients, huge out-of-pocket spending and catastrophic household expenditures. Although China itself is a classic case of an overachiever nation, and has managed to lift more than 800 million of its citizens out of poverty, inequalities in access and affordability of medical care are likely to remain a landmark of most LMICs for decades to come [20].

An increasing number of health economic evaluations in recent years have attempted to capture consequences that extend beyond health outcomes, such as equity and distributional effects [21]. Moreover, new reporting guidelines for health economic evaluation require authors to describe the approaches they used to characterize equity and distributional effects [22]. The WHO Council’s report is likely to provide further impetus to these broader initiatives. Frameworks such as distributional cost-effectiveness analysis, and specific methods for assessing trade-offs between efficiency and equity concerns, such as the equity-efficiency impact plane, are likely to become more prominent in the literature [23]. Nevertheless, it is unclear whether this will extend to local decisions to adjust the cost-effectiveness threshold in favour of socioeconomically disadvantaged groups. There is, however, likely to be greater emphasis placed by the WHO and other specialized agencies of the United Nations on further developing infrastructures and systems that mitigate inequities and generate social cohesion. This process should be facilitated by the willing participation of the national governments of large nations as well as other influential multilateral fora such as the World Health Assembly, World Trade Organization (WTO), the Group of Twenty (G20), Global Health Forum, Global Forum on Human Resources for Health, Health Silk Road as the branch of the One Belt One Road initiative, Council of Europe, and others [24]. Of particular relevance to the research community will be the need for system-level applications of frameworks such as distributional cost-effectiveness analysis to new initiatives that include equity-framed goals.

The other recommendations of the WHO Council’s report are framed around the need for nations to proactively and collaboratively shape markets in order to prioritize human health and wellbeing and planetary health. This includes recommendations to redraw the international architecture of finance to fund health equitably and proactively, including an effective and inclusive crisis response [25]. It also includes the need to build symbiotic public-private alliances that maximize public value, sharing both risks and rewards. Notably, these goals are not to be solely the purview of health ministries, but rather should be the goals for all government agencies. The maximization of human and planetary well-being, rather than maximization of purely economic metrics, such as gross domestic product, should be the ultimate public sector goals.

For this special issue of the journal, we welcome an array of original research studies, reviews and perspective pieces from all involved parties that shine light on the opportunities for achieving the goals enshrined within ‘Economics of Health for All’ and the obstacles to achieving those goals. We welcome research that has a local, national or multi-national focus. Given the scope of *Cost-Effectiveness and Resource Allocation*, we anticipate that several contributions will focus on applications that broaden the range of inputs and outputs of economic evaluation and policy-related research in line with the vision of the WHO Council’s report, and the potential challenges this entails for health care decision-making processes. However, we would welcome contributions that shed light on organizational and system-level factors and processes that facilitate and/or hinder the achievement of health for all.

## Data Availability

Not applicable.
